# High-Fat Feeding Does Not Disrupt Daily Rhythms in Female Mice because of Protection by Ovarian Hormones

**DOI:** 10.3389/fendo.2017.00044

**Published:** 2017-03-14

**Authors:** Brian T. Palmisano, John M. Stafford, Julie S. Pendergast

**Affiliations:** ^1^Department of Molecular Physiology and Biophysics, Vanderbilt University School of Medicine, Nashville, TN, USA; ^2^VA Tennessee Valley Healthcare System, Nashville, TN, USA; ^3^Division of Diabetes, Endocrinology, and Metabolism, Department of Medicine, Vanderbilt University School of Medicine, Nashville, TN, USA; ^4^Department of Biology, University of Kentucky, Lexington, KY, USA

**Keywords:** circadian, C57BL/6J, female, bioluminescence, liver, eating rhythm, high-fat diet, obesity

## Abstract

Obesity in women is increased by the loss of circulating estrogen after menopause. Shift work, which disrupts circadian rhythms, also increases the risk for obesity. It is not known whether ovarian hormones interact with the circadian system to protect females from obesity. During high-fat feeding, male C57BL/6J mice develop profound obesity and disruption of daily rhythms. Since C57BL/6J female mice did not develop diet-induced obesity (during 8 weeks of high-fat feeding), we first determined if daily rhythms in female mice were resistant to disruption from high-fat diet. We fed female PERIOD2:LUCIFERASE mice 45% high-fat diet for 1 week and measured daily rhythms. Female mice retained robust rhythms of eating behavior and locomotor activity during high-fat feeding that were similar to chow-fed females. In addition, the phase of the liver molecular timekeeping (PER2:LUC) rhythm was not altered by high-fat feeding in females. To determine if ovarian hormones protected daily rhythms in female mice from high-fat feeding, we analyzed rhythms in ovariectomized mice. During high-fat feeding, the amplitudes of the eating behavior and locomotor activity rhythms were reduced in ovariectomized females. Liver PER2:LUC rhythms were also advanced by ~4 h by high-fat feeding, but not chow, in ovariectomized females. Together these data show circulating ovarian hormones protect the integrity of daily rhythms in female mice during high-fat feeding.

## Introduction

Disruption of circadian rhythms contributes to obesity and its comorbidities. Circadian rhythms are approximately 24-h fluctuations in physiology and behavior that are synchronized to the environment. In mammals, the circadian system is composed of a network of clocks that are located in nearly every tissue in the body. The master circadian clock in the suprachiasmatic nucleus (SCN) in the brain receives information about the timing of the environmental light–dark cycle and in turn coordinates the timing (or phases) of the other clocks located throughout the body (1, 2). Numerous epidemiological studies of shift workers as well as laboratory studies of healthy people showed that disruption of the circadian system increases the risk for obesity and metabolic dysfunction (3–10).

Animal studies have probed the mechanisms underlying the reciprocal interactions between the circadian system and metabolism. Disabling the molecular timekeeping mechanism of circadian clocks in rodents altered glucose regulation and caused obesity (11–14). And, conversely, diet-induced obesity disrupted the circadian system (15–19). We and others found the daily rhythm of eating behavior was altered during high-fat feeding such that mice ate across the day instead of eating mostly at night, which is the normal feeding time for rodents (15, 16). This disrupted eating rhythm was a determinant of obesity since restricting high-fat diet feeding only to the nighttime inhibited obesity (20, 21). We also previously showed that high-fat feeding disrupted the temporal coordination between the timing of body clocks by altering the phase of the liver circadian clock (16).

The drawback of these previous animal studies, including our own, was they were performed exclusively in male animals. This is problematic because obesity and its related complications develop differently in men and women. Pre-menopausal women are protected from the negative consequences of obesity such as the metabolic syndrome (22). However, the loss of estrogen after menopause increases the risk of life-threatening, obesity-related complications such as cardiovascular disease and stroke (22–24). To our knowledge, no study has investigated the integrity of metabolic circadian rhythms in females or the role of ovarian hormones in regulating high-fat diet-induced disruption of daily rhythms. In this study, we addressed these questions by investigating daily rhythms in intact and ovariectomized female mice during high-fat feeding.

## Materials and Methods

### Animals

Heterozygous C57BL/6J PERIOD2:LUCIFERASE (PER2:LUC) (2) and wild-type littermate (N23 to 25 generations of backcrossing with C57BL/6J mice, Jackson Laboratory, Bar Harbor, ME, USA) mice were born and raised in 12-h light/12-h dark (12L:12D; light intensity ~350 lux) at Vanderbilt University. At weaning (21 days old), mice were group housed (2–4 mice/cage). Genotype was determined by measuring bioluminescence from tail snips from 21-day-old mice. All mice (breeders and pups) were fed chow (13.5% kcal from fat, LabDiet 5L0D) *ad libitum* until they underwent experimental diet manipulations. All procedures were conducted in accordance with the guidelines of the National Institutes of Health Guide for the Care and Use of Laboratory Animals and were approved by the Institutional Animal Care and Use Committee at Vanderbilt University (protocol number M/13/081).

### Experimental Protocols

#### Experiment I. Effect of Chronic High-Fat Diet Consumption on Body Weight in Female Mice

Heterozygous PER2:LUC C57BL/6J and wild-type female mice were single housed in cages (33 cm × 17 cm × 14 cm) with locked running wheels (wheels could not rotate) in light-tight boxes in 12L:12D (light intensity 200–300 lux; temperature inside light-tight boxes: 25.5 ± 1.5°C) at 7 weeks old and maintained on chow *ad libitum*. Beginning at 8 weeks old, mice were fed either chow or 45% high-fat diet (Research Diets D01060502) for 8 weeks. Body weight was measured weekly (always within 3 h before lights off).

#### Experiment II. Effects of Acute High-Fat Diet Consumption on Circadian Organization and the Eating Behavior and Locomotor Activity Rhythms in Female Mice

Heterozygous PER2:LUC C57BL/6J female mice were single housed in cages with locked running wheels at 7 weeks old in light-tight boxes in 12L:12D and fed chow. Body weight and food intake were measured weekly (always within 3 h before lights off). Locomotor activity and eating behavior were continuously measured. Beginning at 8 weeks old, mice were fed either chow or 45% high-fat diet for 1 week. At 9 weeks old, tissues were explanted and cultured to measure bioluminescence rhythms.

#### Experiment III. Effect of Ovariectomy in Mediating High-Fat Diet Effects on Daily Rhythms

Heterozygous PER2:LUC C57BL/6J female mice were ovariectomized at 6 weeks old and single housed following the surgery in light-tight boxes in 12L:12D. Body weight and food intake were measured weekly (always within 3 h before lights off). Locomotor activity and eating behavior were continuously measured. Mice were fed chow *ad libitum* for 2 weeks. Beginning at 8 weeks old, mice were fed either chow or 45% high-fat diet for 1 week. At 9 weeks old, livers were explanted, cultured, and bioluminescence rhythms were measured.

### Bioluminescence Recording and Analysis

Within 1.5 h before lights out, tissue explants were prepared as previously described (25). In the first experiment (Figure S1 in Supplementary Material), SCN, arcuate complex, pituitary, liver, lung, aorta, spleen, and white adipose tissue were collected from each mouse and cultured as previously described (16). In the subsequent experiments in intact and ovariectomized mice, only liver explants were cultured from female mice. Bioluminescence was measured with the LumiCycle in 10-min intervals (Actimetrics Inc., Evanston, IL, USA). The data were detrended (by subtracting the 24 h moving average) and smoothed (0.5 h adjacent average) using LumiCycle software. Then ClockLab analysis software was used to determine the phase (peak of bioluminescence occurring between 12 h and 36 h in culture) of PER2:LUC expression.

### Behavior Recording and Analysis

General locomotor activity data were collected every minute using passive infrared sensors (sensors record a maximum of one count every 6 s; model 007.1, Visonic LTD). Double-plotted actograms of locomotor activity were created with Clocklab (6-min bins; scaled setting). Cosinor analysis was performed for each mouse on activity profiles of 5 days of chow feeding or 5 days of high-fat feeding with Clocklab software. Cosinor analysis fits a cosine curve to the time series data and determines the amplitude (half of the peak-to-trough value), mesor (midline or rhythm-adjusted mean), and acrophase (the timing of the peak of the rhythm) of the rhythm (period was 24 h) (27). Mean activity profiles were generated by averaging daily locomotor activity in 6-min bins during either chow or high-fat feeding for all mice.

Eating behavior was continuously recorded using an infrared video camera (PYLE PLCM22IR Flush Mount Rear View Camera with 0.5 lux Night Vision, Pyle Audio Inc., Brooklyn, NY, USA) interfaced to a computer with VideoSecu4 (16). Eating behavior was analyzed in 1-min bins (coded as 1 for eating behavior and 0 for no eating behavior) as previously described (16). Eating behavior data were plotted in circular histograms and analyzed with circular statistics (Oriana 4.0; Kovach Computing Services, Wales, UK). Circular histograms show the distribution of eating events across the day (lights on 0–12). Circular statistics were used to determine the vector of the rhythm. Grand mean vectors describe the phase (direction) and amplitude (length) of the mean rhythms of female mice (*n* = 5) during chow (day 7) or high-fat diet (days 9 and 14) feeding.

### Ovariectomy Surgery

At 6 weeks of age, female mice were ovariectomized as described previously (28). Briefly, animals were anesthetized under inhaled isoflurane and administered analgesic pre-operatively. After midline dorsal skin incision, two lateral incisions of the dorsal peritoneal wall were made and ovaries were removed. Peritoneal incisions were closed with single simple interrupted stiches and the skin incision was closed with autoclips. Mice were housed individually following surgery and allowed to recover for 7 days prior to study.

### Statistical Analyses

Two-way Repeated Measures ANOVA (with *post hoc* Fisher LSD test) was used to determine if body weight was affected by high-fat diet compared to chow consumption over time and to determine the effects of sex, diet, and ovariectomy on eating behavior rhythms (OriginPro 2016, Northhampton, MA, USA). Independent *t*-tests (two-tailed) were used to compare the phases of liver PER2:LUC rhythms in intact or ovariectomized mice (OriginPro 2016). Paired *t*-tests (two-tailed) were used to compare the amplitudes, phases, and mesors of the locomotor activity rhythms. Circular data were plotted and analyzed using Oriana 4.0. The mean vector of each day of behavior data (for individual mice) was determined by Rayleigh’s uniformity test to indicate the angle (μ) and degree of clustering (length; *r*). Grand mean vectors (to analyze groups of mice) were analyzed using Hotelling’s one sample test. The length of the vector describes the uniformity of the distribution of activity such that short vectors indicate that activity is more evenly distributed across the cycle. Significance was ascribed at *p* < 0.05.

## Results

### Female C57BL/6J Mice Are Resistant to Diet-Induced Obesity

**Figure 1:**
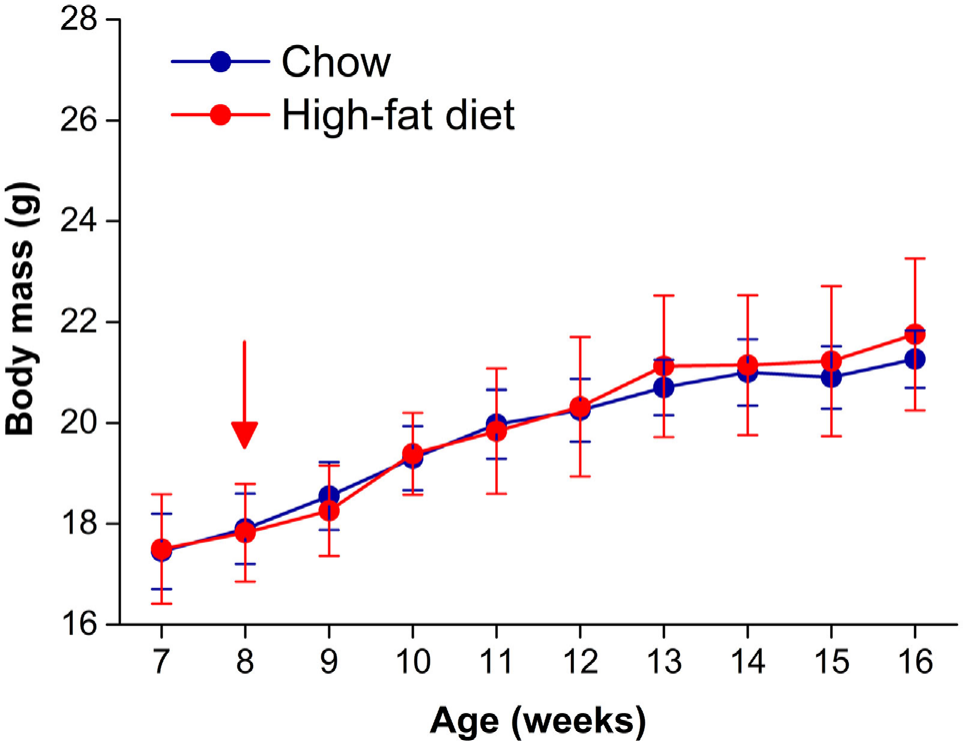
**Female C57BL/6J mice are resistant to diet-induced obesity**. Body masses (mean grams ± SD) of female C57BL/6J mice fed either chow (blue circles, *n* = 8) or 45% high-fat diet (red circles, *n* = 9) for 8 weeks. High-fat feeding began at 8 weeks old (at red arrow).

Male C57BL/6J mice become obese when they consume high-fat diet (29, 30). We first tested whether female C57BL/6J mice fed 45% high-fat diet developed diet-induced obesity. We fed female mice chow or high-fat diet for 8 weeks and measured body weight weekly (Figure 1). Although all mice gained weight over the 8-week experiment (time: *F* = 40.83, *p* < 0.001), there was no significant interaction between diet and time (*F* = 2.6, *p* = 0.11). Therefore, female mice were resistant to diet-induced weight gain.

### High-Fat Feeding Does Not Alter Molecular Timekeeping Rhythms in Tissues in Female Mice

We previously found that consumption of high-fat diet disrupted the temporal relationship between tissue molecular rhythms in male mice by advancing the phase of the liver circadian clock rhythm (16). Thus, we next determined the effects of high-fat diet consumption on circadian rhythms in central and peripheral tissues in female mice. PERIOD2 is a component of the molecular timekeeping mechanism of the circadian clock and is a target gene of the Clock/Bmal1 transcription factor network (31, 32). In PER2:LUC mice, the luciferase gene is knocked in to the 3′ end of the *Period2* locus resulting in the expression of the PER2:LUC fusion protein (2). We assessed the molecular circadian clock rhythm by measuring bioluminescence from tissues explanted from PER2:LUC reporter mice (2).

**Figure 2:**
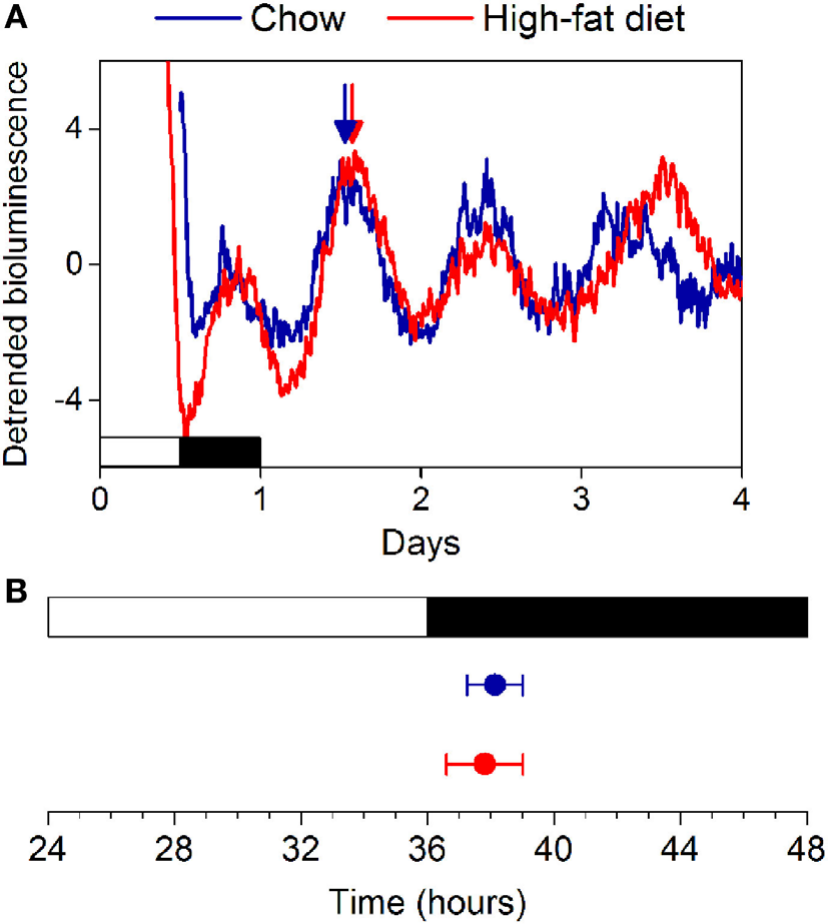
**High-fat diet does not alter the phase of the liver circadian clock in female mice**. **(A)** Female C57BL/6J PER2:LUC mice were fed chow (blue circles) or high-fat diet (red circles) for 1 week and liver explants were cultured. **(B)** The mean phases (±SD) of the peaks of the bioluminescence rhythms were plotted relative to last lights on (12L:12D cycle indicated by white and black bars, respectively). Number of livers/total number cultured: Chow 7/7; High-fat diet: 5/7 (*p* = 0.60).

We fed female mice either chow or high-fat diet for 1 week and measured rhythms of PER2:LUC bioluminescence in explanted tissues (Figure S1 in Supplementary Material). Similar to our previous study in males, we found that the phases of the PER2:LUC rhythms in the SCN, pituitary, lung, aorta, spleen, arcuate nucleus, and white adipose tissue were not affected by high-fat diet consumption. We also found that the phase of the liver PER2:LUC rhythm was not affected by high-fat feeding in female mice (Figure S1 in Supplementary Material). This surprisingly contrasted with our previous finding that the phase of the liver PER2:LUC rhythm was advanced by ~5 h in male mice consuming high-fat diet (16). We therefore repeated the experiment in female mice, cultured only livers, and confirmed the phase of the liver PER2:LUC rhythm was not altered by 1 week of high-fat diet consumption (Figure 2: *p* = 0.60).

### The Eating Behavior Rhythm Is Not Affected by High-Fat Feeding in Female Mice

**Figure 3:**
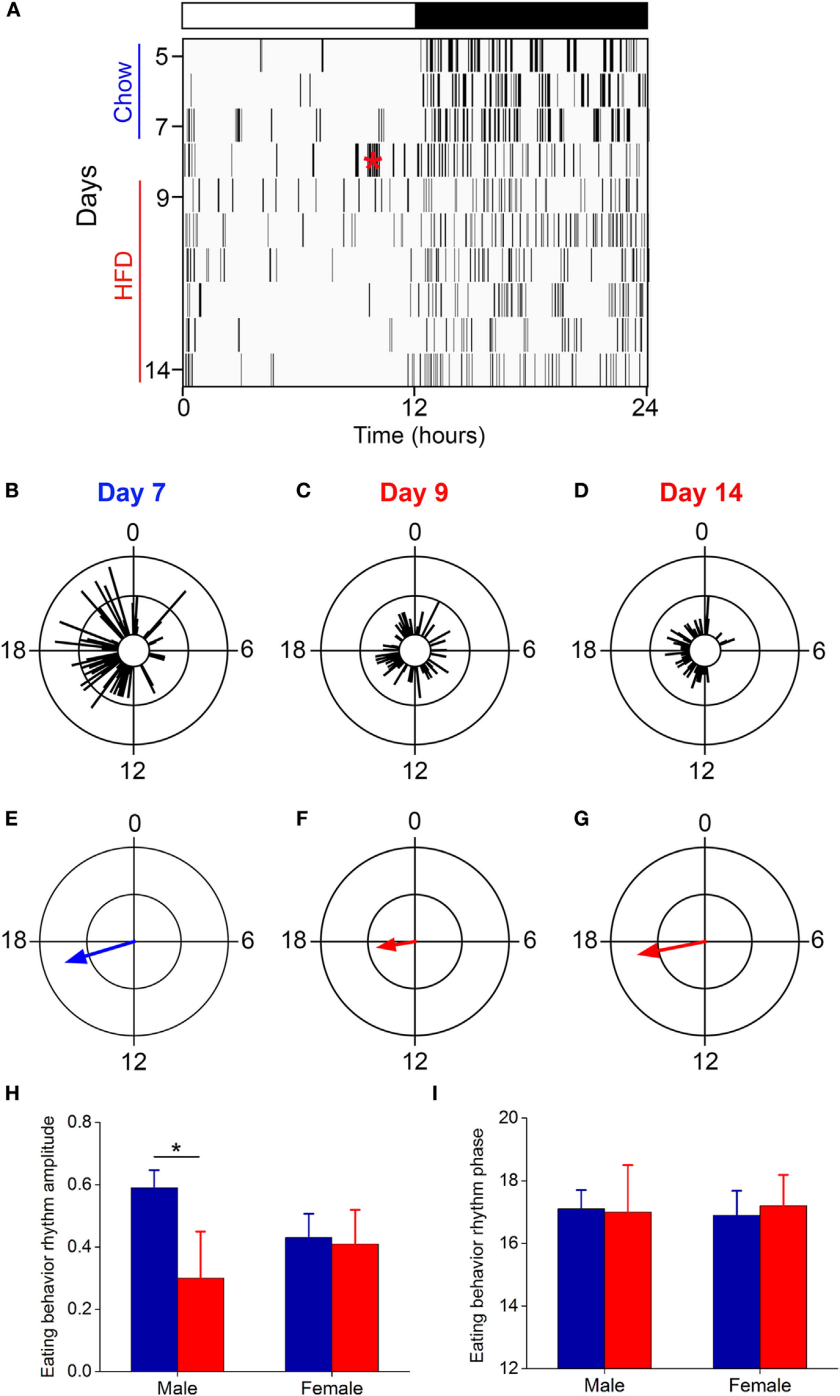
**The eating behavior rhythm is robust in females fed high-fat diet**. Eating behavior was measured with infrared video cameras. **(A)** Representative actogram of eating behavior (1-min bins) of a female mouse fed chow (days 1–7, blue) and then switched to 45% high-fat diet (days 9–14, red, HFD added at red asterisk on day 8). Each vertical line is an eating event (1-min bins). Representative circular histograms show the distribution of eating behavior across the day (10-min bins) in an individual mouse during one day of chow [**(B)**: day 7], during the first day of HFD [**(C)**: day 9], and during the sixth day of HFD feeding [**(D)**: day 14]. Scale: inner circle, 0; middle circle, 5; outer circle, 10. Grand mean vectors of eating behavior show the average eating behavior of female mice (*n* = 5) during chow [**(E)**: day 7] and HFD [**(F)**: day 9; **(G)**: day 14] feeding. Scale: inner circle, 0; middle circle, 0.3; outer circle, 0.6. Lights were on from 0 to 12. Circular statistics are shown in Table S1 in Supplementary Material. Mean (±SD) amplitudes [**(H)**, *y*-axis: length of grand mean vector] and phases [**(I)**, *y*-axis: phase in ZT of grand mean vector] of male (*n* = 5) and female (*n* = 5) mice. Male data were taken from our previous study (26).

Several studies, including our own, have shown that the amplitude of the daily rhythm of eating behavior was markedly reduced or eliminated during high-fat diet consumption in male C57BL/6J mice (15, 16, 26, 33). We examined the eating behavior rhythm in females using an infrared video camera (Figure 3; Figure S2 in Supplementary Material; data from all mice shown in Figure S3 in Supplementary Material). During chow feeding, females had a robust, high-amplitude eating behavior rhythm characterized by a few snacks during the day and the majority of eating during the night [a pattern indistinguishable from chow-fed males (16)] (Figure 3A: days 5–7; Figures 3B,E). During the first 24 h of high-fat diet feeding, females exhibited continuous eating behavior, resulting in a low-amplitude eating behavior rhythm, which is also similar to males on high-fat diet (16, 26, 33) (Figure 3A: day 9; Figures 3C,F). However, by 1 week of high-fat feeding, females had high-amplitude robust daily rhythms of eating behavior (Figure 3A: days 11–14; Figures 3D,G). Thus, females displayed the novelty response to palatable high-fat diet, but this response extinguished and their chow-like eating behavior rhythm returned. These data demonstrate that high-fat diet was not aversive to females. In fact, caloric intake increased 15% during 1 week of high-fat feeding, but the females did not gain more weight than chow-fed controls (Figure S2 in Supplementary Material; caloric intake did not increase during chow feeding). Moreover, females had fewer eating events (Figure S4A in Supplementary Material: intact) and ate fewer grams of food (Figure S4B in Supplementary Material: intact) during high-fat feeding compared to chow. These data suggest that the females had the appropriate homeostatic response to the calorie-dense high-fat diet by reducing the mass of food eaten in an attempt to scale down caloric intake to constrain their body weight gain.

We next compared the amplitudes and phases of the eating behavior rhythms between males [data from our previous study using an identical protocol (26)] and females during chow and high-fat feeding (Figures 3H,I). There was a significant interaction of sex and diet (*F* = 12.3, *p* = 0.02) on the amplitudes of the eating behavior rhythms (Figure 3H). Compared to chow, 1 week of high-fat feeding significantly reduced the amplitude of the eating behavior rhythm in males (*p* = 0.04), but not females (*p* = 0.99). There were no significant effects of sex and/or diet on the phase of the eating behavior rhythms (Figure 3I). Thus, although female mice initially responded to high-fat diet with disrupted low-amplitude eating behavior, within 1 week female mice, unlike males, reverted to the high-amplitude eating rhythm of chow-fed mice.

### The Amplitude of the Locomotor Activity Rhythm Is Not Affected by High-Fat Feeding in Female Mice

**Figure 4:**
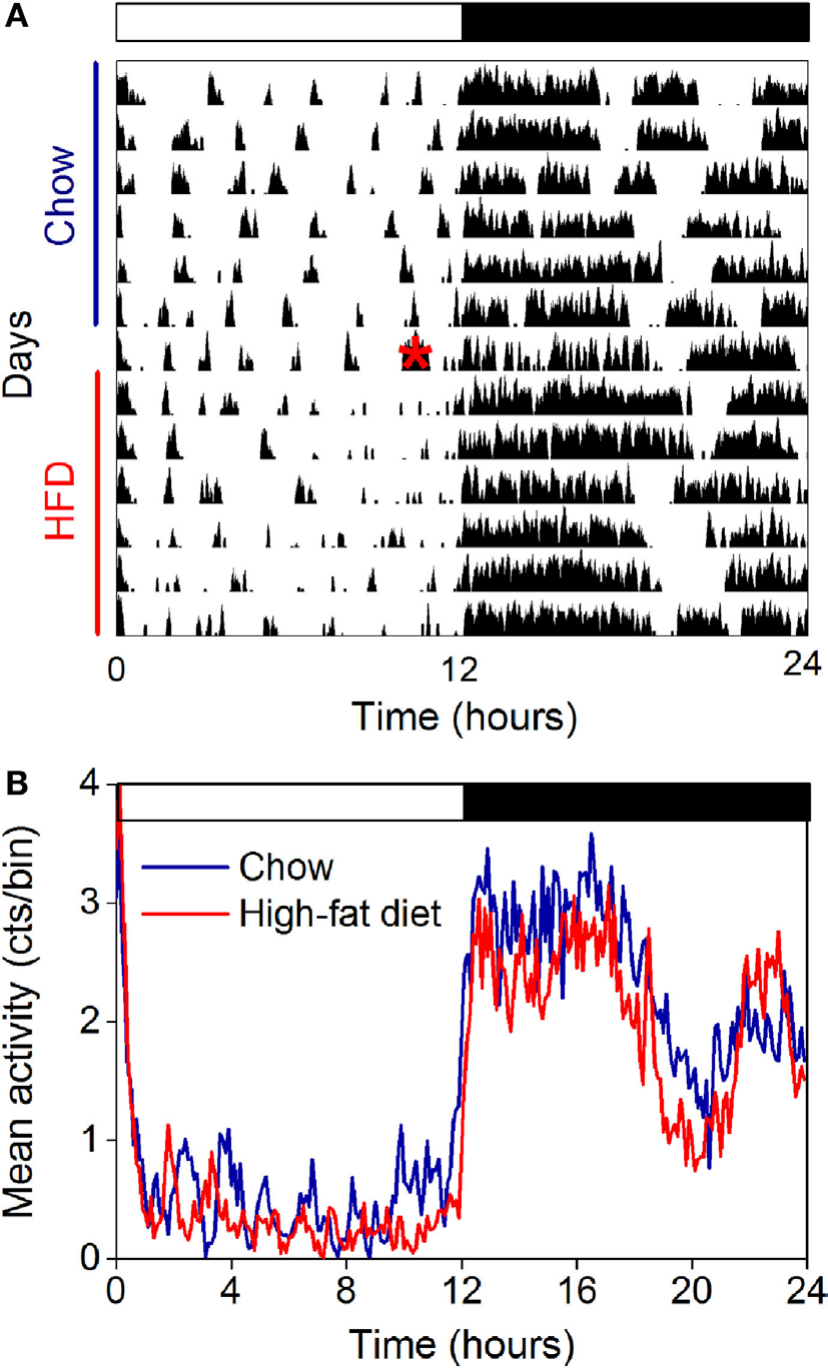
**The amplitude of the locomotor activity rhythm in females is not affected by high-fat feeding**. Locomotor activity was measured with passive infrared sensors. **(A)** Representative actogram of locomotor activity (6-min bins; scale: 5) of a female mouse fed chow (days 1–7) and then switched to 45% high-fat diet (days 9–14, high-fat diet added at red asterisk on day 8). **(B)** Group average activity profiles (*y*-axis: counts/6 min bin) of females fed chow (blue) for 1 week and then high-fat diet (red) for 1 week (*n* = 5 mice).

The amplitude of the locomotor activity rhythm is reduced in male C57BL/6J mice (15, 18, 33). Thus, we next measured the locomotor activity rhythm with infrared motion sensors in female mice fed chow for 1 week and then high-fat diet for 1 week (Figure 4, actograms from all mice shown in Figure S5 in Supplementary Material; *n* = 5). In contrast to male mice, we found the amplitude and phase of the locomotor activity rhythm were not affected by high-fat feeding (Figure 4; Table 1). The mesor, or mean level of activity, was reduced slightly during high-fat feeding compared to chow feeding (Table 1).

**Table 1:** **Cosinor analysis of locomotor activity rhythms in intact female mice**.

	Amplitude	Phase	Mesor
Chow	2.5 ± 1.1	17.2 ± 0.4	1.5 ± 0.6
High-fat diet	2.2 ± 1.1	18.0 ± 0.6	1.3 ± 0.6
*p*	0.19	0.06	0.03

### Ovariectomy Abolishes Protection of Daily Rhythms from High-Fat Diet Feeding

**Figure 5:**
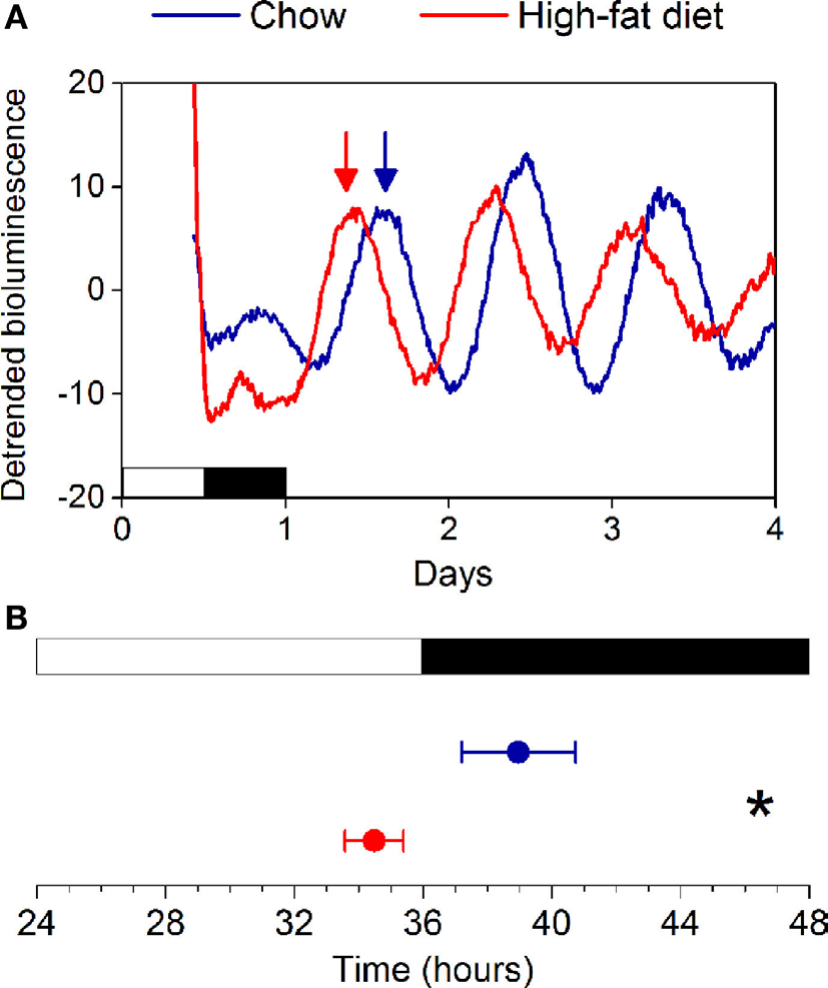
**High-fat diet feeding advances the phase of the liver clock in ovariectomized females**. **(A)** Representative traces of PER2:LUC bioluminescence recorded from ovariectomized mice fed chow (blue) or 45% high-fat diet (red) for 1 week (*y*-axis: counts per second). Phases were determined from the peaks of bioluminescence occurring between 12 and 36 h in culture (indicated by arrows). **(B)** Mean phases (±SD) of liver PER2:LUC rhythms from chow (*n* = 6)- and high-fat diet (*n* = 6)-fed ovariectomized mice (*p* < 0.001).

We and others have previously shown that female mice are susceptible to diet-induced obesity after ovariectomy (34–37). Thus, we next determined if ovarian hormones were required to confer protection of daily rhythms from high-fat feeding in females. We ovariectomized female mice and then fed them chow or high-fat diet for 1 week (Figure S6 in Supplementary Material). In contrast to intact females, the phase of the liver PER2:LUC rhythm was advanced ~4 h in ovariectomized mice fed high-fat diet compared to those fed chow (Figure 5).

**Figure 6:**
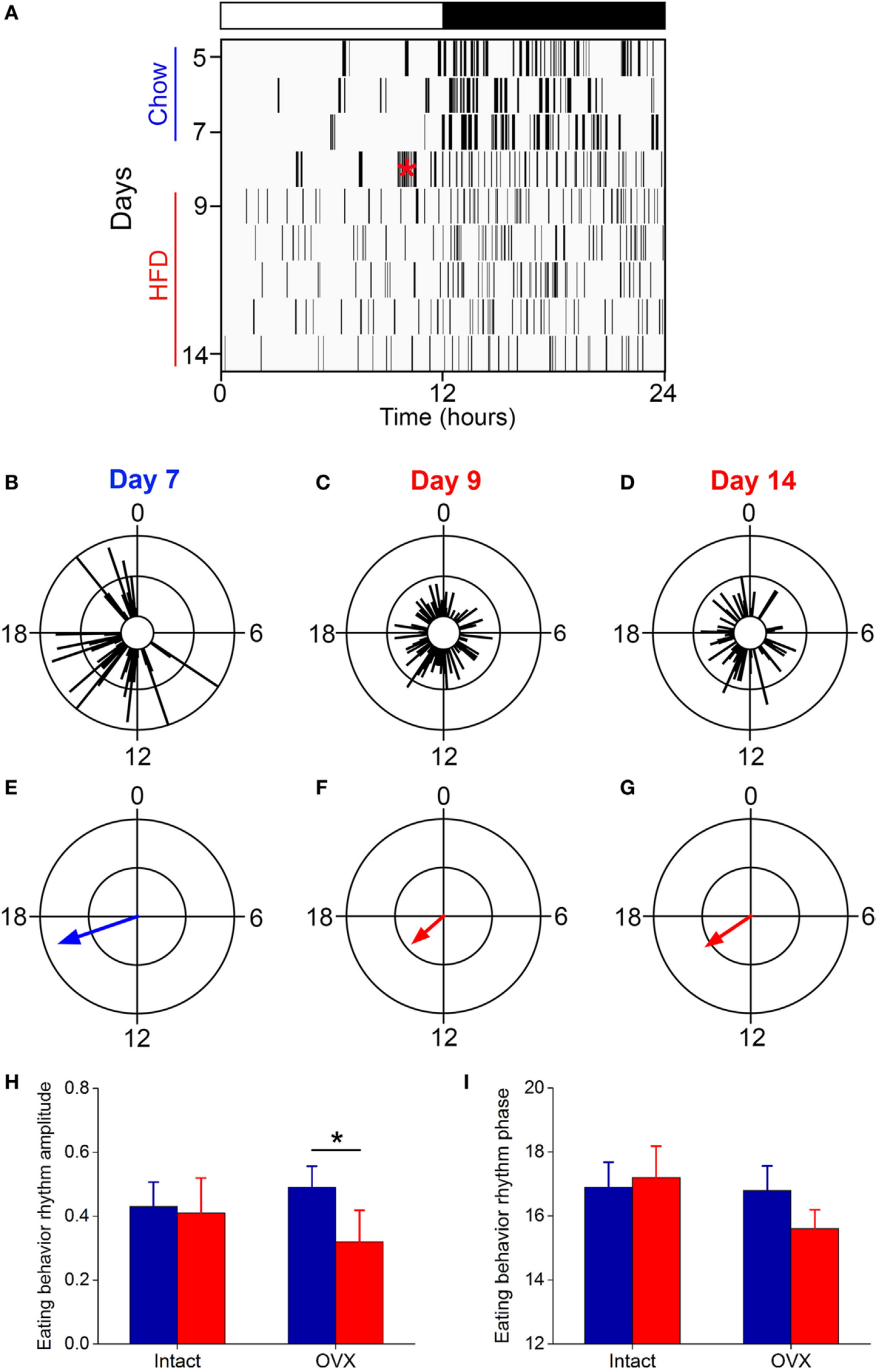
**The eating behavior rhythm is compromised in ovariectomized females fed high-fat diet**. Eating behavior was measured with infrared video cameras. **(A)** Representative actogram of eating behavior (1-min bins) of an ovariectomized female mouse fed chow (days 1–7, blue) and then switched to 45% high-fat diet (days 9–14, red, high-fat diet added at red asterisk on day 8). Each vertical line is an eating event. Representative circular histograms show the distribution of eating behavior across the day (10-min bins) in an individual ovariectomized female mouse during 1 day of chow [**(B)**: day 7], during the first day of HFD [**(C)**: day 9], and during the sixth day of HFD feeding [**(D)**: day 14]. Scale: inner circle, 0; middle circle, 5; outer circle, 10. Grand mean vectors of eating behavior show the average eating behavior of ovariectomized female mice (*n* = 5) during chow [**(E)**: day 7] and HFD [**(F)**: day 9; **(G)**: day 14] feeding. Scale: inner circle, 0; middle circle, 0.3; outer circle, 0.6. Lights were on from 0 to 12. Circular statistics are shown in Table S1 in Supplementary Material. Mean (±SD) amplitudes [**(H)**, *y*-axis: length of grand mean vector] and phases [**(I)**, *y*-axis: phase in ZT of grand mean vector] of intact (*n* = 5; data from Figure 3) and ovariectomized (*n* = 5) female mice.

We next measured daily rhythm of eating behavior (Figure 6) in ovariectomized females fed chow or high-fat diet. Ovariectomized females fed chow had robust, high-amplitude eating behavior rhythms (Figure 6A: days 5–7; Figures 6B,E; all mice shown in Figure S7 in Supplementary Material). The amplitude of the eating behavior rhythm was reduced by high-fat feeding in ovariectomized females such that eating events were spread across the day and night (Figure 6A: days 9–14; Figures 6C,D,F,G; all mice shown in Figure S7 in Supplementary Material). The low-amplitude eating rhythm persisted 7 days after high-fat diet was introduced (Figures 6D,G). When we compared the amplitudes of the eating behavior rhythms between intact and ovariectomized females, there was a significant interaction of ovariectomy and diet (*F* = 9.2, *p* = 0.04) on the amplitudes of the eating behavior rhythms (Figure 6H). Compared to chow, 1 week of high-fat feeding significantly reduced the amplitude of the eating behavior rhythm in ovariectomized females (*p* = 0.04), but not intact females (*p* = 0.74). There were no significant effects of ovariectomy and diet on the phase of the eating behavior rhythm (*p* = 0.06). Ovariectomized females ate more calories during high-fat feeding compared to chow feeding (Figure S6 in Supplementary Material). Similar to intact females, ovariectomized mice had fewer eating events (Figure S4A in Supplementary Material: OVX) and ate fewer grams of food (Figure S4B in Supplementary Material: OVX) during high-fat feeding compared to chow.

**Figure 7:**
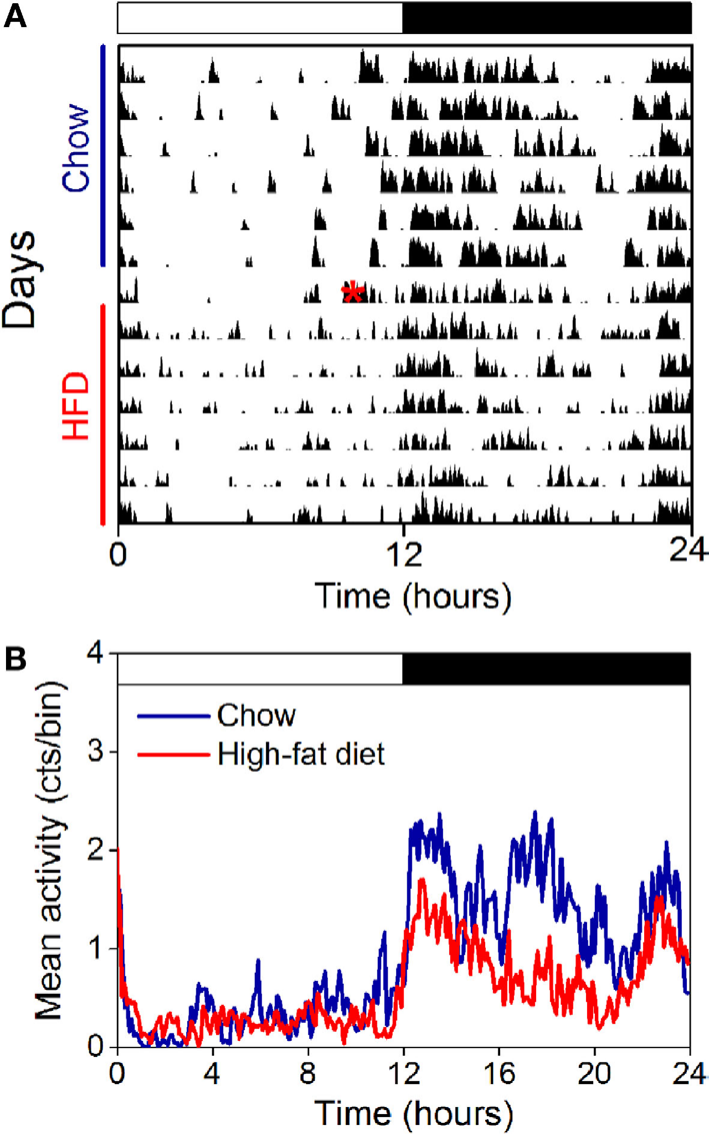
**The locomotor activity rhythm is reduced by high-fat feeding in ovariectomized female mice**. Locomotor activity was measured with passive infrared sensors. **(A)** Representative actogram of locomotor activity (6-min bins; scale: 5) of an ovariectomized female mouse fed chow (days 1–7) and then switched to 45% high-fat diet (days 9–14, high-fat diet added at red asterisk on day 8). **(B)** Group average activity profiles (*y*-axis: counts/6-min bin) of ovariectomized females fed chow (blue) for 1 week and then high-fat diet (red) for 1 week (*n* = 5 mice).

We also measured the locomotor activity rhythm in ovariectomized females fed chow and high-fat diet (Figure 7). Immediately upon addition of high-fat diet, consolidated bouts of locomotor activity dissipated into shorter activity bouts (Figure 7A). This effect of high-fat diet persisted for the entire week of high-fat feeding. The amplitude and mesor (mean) of the locomotor activity rhythm were also reduced during high-fat feeding in ovariectomized females (Figure 7B; Table 2). There were no significant effects of ovariectomy and/or diet on the phase of the locomotor activity rhythm.

**Table 2:** **Cosinor analysis of locomotor activity rhythms in ovariectomized female mice**.

	Amplitude	Phase	Mesor
Chow	1.6 ± 0.6	17.0 ± 0.7	0.9 ± 0.4
High-fat diet	0.6 ± 0.1	17.1 ± 0.8	0.6 ± 0.2
*p*	0.02	0.77	0.06

## Discussion

Obesity and metabolic dysfunction are linked to disruption of circadian rhythms in human and animal studies. Studies in male mice have shown that high-fat feeding alters tissue, hormone, and behavior (eating and locomotor activity) rhythms (15–18). The disruption of daily rhythms in male mice is accompanied by development of profound obesity. However, the effects of high-fat feeding on daily metabolic rhythms in females have not been investigated. In this study, we showed that, in contrast to male mice, daily rhythms in females are protected from disruption by high-fat feeding.

Molecular rhythms of circadian gene expression and metabolites are altered in the livers of male mice fed high-fat diet acutely and chronically (15, 16, 19, 26, 33). We have postulated that temporal misalignment of the liver circadian clock and its rhythmic outputs are determinants of obesity. The current study further supports this hypothesis. We found the phase of the liver circadian clock is not affected by high-fat feeding in female mice, which are also resistant to diet-induced obesity. After ovariectomy, female mice are susceptible to diet-induced obesity (34, 35, 37), and the phases of their liver molecular clocks are markedly altered (liver phase is advanced 4 h). It is possible that circulating estrogen, or lack thereof, directly alters the circadian clock in the liver. Indeed, estradiol has been shown to alter the PER2:LUC rhythms in cultures of explanted uteruses and kisspeptin neurons(38, 39). Moreover, estrogen receptor signaling in the liver is incredibly responsive to estrogen *in vivo* (40). In future studies, we will investigate the mechanism whereby estrogen or other ovarian hormones regulate the phase of the liver circadian clock.

Disruption of the eating behavior rhythm contributes to the development of diet-induced obesity in males (20). When given high-fat diet, the amplitude of the eating rhythm is markedly reduced such that male mice eat throughout the 24-h day (light and dark phases) (15, 16). Restricting high-fat feeding to only the nighttime, which is when mice consume most of their calories, protects males from diet-induced obesity (20). In contrast to males, we found that females were resistant to the effects of high-fat diet on daily rhythms. Thus, in females, the daily rhythm of eating behavior in female mice is robust during high-fat feeding such that eating is consolidated during the nighttime. This robust eating behavior rhythm during high-fat feeding is lost after ovariectomy. Thus, ovarian hormones play a critical role in maintaining circadian feeding behavior rhythms despite high-fat diet feeding in female mice.

Our study also suggests that ovarian hormones differentially regulate the effects of high-fat feeding on the daily eating rhythm and homeostatic regulation of caloric intake. Ovariectomy abolished protection of the eating rhythm from high-fat feeding. In contrast, both intact and ovariectomized females had reduced eating events and food intake (measured in grams of food consumed) during high-fat feeding compared to chow. Thus, in ovariectomized mice, the homeostatic reduction in food intake is intact, since the mice eat significantly less of the calorie-dense high-fat diet compared to chow (Figure S4 in Supplementary Material), while the daily eating rhythm is markedly altered.

The role of ovarian hormones in maintaining circadian rhythms may be critical in protecting females from diet-induced obesity. It is likely that estrogen plays a major role in regulating circadian eating behavior during high-fat feeding. Previous studies have shown that estrogen acutely reduces feeding and the daily rhythm of food intake varies across the estrous cycle in rats (41, 42). In this study, we did not control for the estrous cycle in the intact females. It will be interesting to determine in future studies if the amplitude of the eating behavior rhythm during high-fat feeding fluctuates with the stage of the estrous cycle. In future studies, we will treat ovariectomized females with estrogen to determine if this restores protection of daily rhythms during high-fat feeding.

Studies in rodents and humans have demonstrated the roles of estrogen in controlling energy homeostasis and glucose metabolism [for review, see Ref (43)]. While the prevalence of obesity is equivalent in males and pre-menopausal women, obesity prevalence increases sharply in post-menopausal women (42%) (44). Estrogen deficiency after menopause predisposes women to obesity, the metabolic syndrome, and type 2 diabetes (22). Likewise, ovariectomy increases adiposity in rodents, and this increase in adiposity is prevented by estrogen replacement (41, 45). Thus, estrogens play an important role in energy homeostasis in both humans and rodents. We hypothesize that estrogen may play a role in protecting daily rhythms from the effects of high-fat feeding in female mice since ovariectomy abolished this protection. Alternatively, other ovarian hormones, such as progesterone, may confer protection of rhythms from high-fat feeding. Future studies will determine whether estrogen or other ovarian hormones are responsible for this protection.

The SCN controls the daily rhythm of food intake (46). It is possible that estrogen acts directly on SCN neurons to control the eating behavior rhythm. However, we speculate instead that estrogen acts downstream of the SCN to protect the daily rhythm of eating behavior from high-fat feeding. The reasons are twofold. First, the circadian rhythm in the SCN and its outputs are only moderately, at best, altered by high-fat feeding (15, 16, 18). This is in contrast to our observation that the eating behavior rhythm is all but eliminated during high-fat feeding in male mice and ovariectomized female mice (16). Second, the circadian rhythm in the SCN and its outputs are only moderately affected by manipulations of estrogen signaling (47). Moreover, estrogen receptors are expressed in few cells in the SCN (48). Taken together, we propose that estrogen acts outside of the SCN, perhaps in other feeding-related hypothalamic nuclei, to modulate the eating behavior rhythm during high-fat feeding.

High-fat feeding reduces the amplitude of the locomotor activity rhythm in male C57BL/6J mice (15, 18, 33). In contrast to males, the amplitude of the locomotor activity rhythm in female mice was not altered by high-fat feeding. Consistent with a previous study, the amplitude of the activity rhythm was reduced by ovariectomy in chow-fed mice (49). Additionally, in the absence of ovarian hormones, the amplitude of locomotor activity was reduced by half during high-fat feeding relative to chow feeding. Thus, ovarian hormones were also required to protect the daily activity rhythm. Similar to previous studies in males (15, 18), high-fat diet feeding reduced the mean activity level in both intact and ovariectomized females. Estrogen could act directly on SCN neurons or outside of the SCN to regulate the amplitude of the locomotor activity rhythm. It is difficult, if not impossible, to address this question because there is currently no way to eliminate the function of estrogen receptors exclusively in SCN cells (e.g., there is no Cre driver that is exclusively expressed in SCN cells).

Diet-induced obesity studies in rodents have provided promising avenues for “repairing” circadian disruption and improving metabolism. For example, restricting eating to specific times of day, called time-restricted feeding, is gaining traction as a plausible behavioral strategy to reduce obesity (50). However, while proof-of-concept for this therapy is strong in studies of male mice (20), our study suggests this therapy may not be effective in females. This study highlights the necessity of studying female models of diet-induced obesity when developing and testing novel therapeutics.

The current study elucidates protection of daily rhythms as a putative mechanism whereby females are resistant to diet-induced obesity. Notably, daily rhythms in females, compared to males, respond oppositely to high-fat feeding. Understanding the circadian mechanisms conferring obesity and designing therapeutics will employ distinct approaches in males and females.

## Author Contributions

BP performed experiments and participated in the discussion and writing of the manuscript. JS contributed to the design of the experiments and participated in discussion and writing of the manuscript. JP designed and performed experiments, analyzed data, and wrote the manuscript.

## Conflict of Interest Statement

The authors declare that the research was conducted in the absence of any commercial or financial relationships that could be construed as a potential conflict of interest.
