# Supplementary material for: High-Fat Feeding Does Not Disrupt Daily Rhythms in Female Mice because of Protection by Ovarian Hormones
Source: Front Endocrinol (Lausanne). 2017 Mar 14;8:44. doi: 10.3389/fendo.2017.00044 (PMC5348546; doi:10.3389/fendo.2017.00044)
Supplement: Supplementary file 1 [file Data_Sheet_1.PDF]

Supplemental Information:
High-fat feeding does not disrupt daily rhythms in female mice because of protection by ovarian hormones

Brian T. Palmisano, John M. Stafford, Julie S. Pendergast

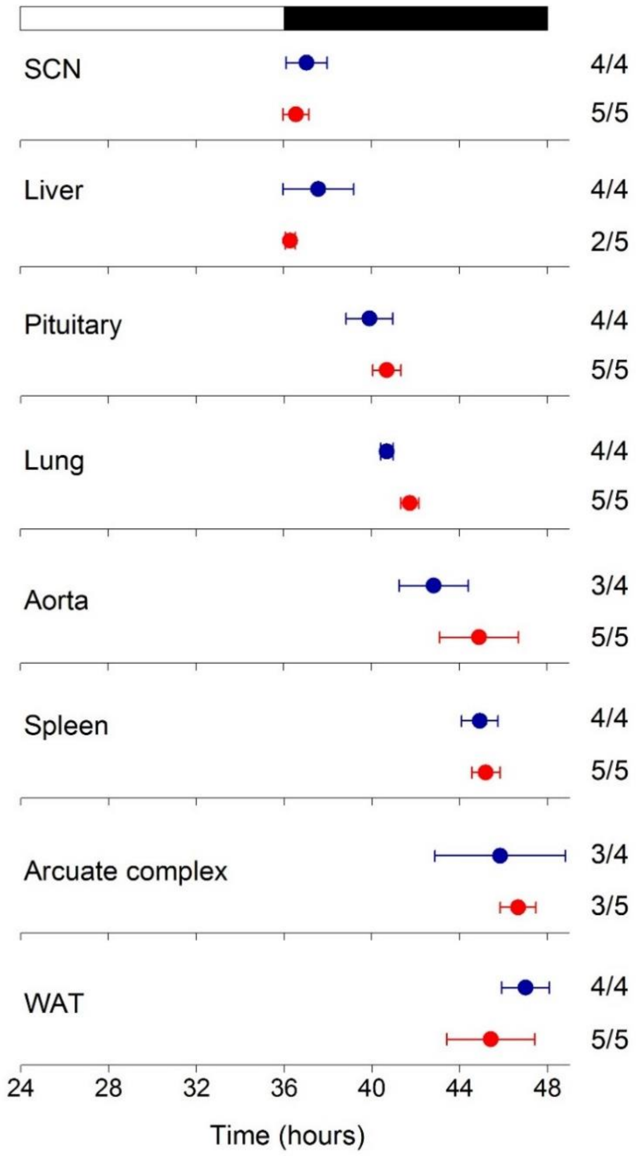

Figure S1. Tissue molecular timekeeping rhythms are not altered by high-fat feeding in female mice. Single-housed female heterozygous PER2::LUC mice were fed chow (blue symbols) or high-fat diet (red symbols) for 1 week. The mean (±SD) phases were determined from the peaks of PER2::LUC expression during the interval between 12 and 36 hours in culture and were plotted relative to the time of last lights on where 24h is lights on and 36h is lights off (white and black bar at top). The sample size is shown (number of rhythmic tissues/number of tissues tested).

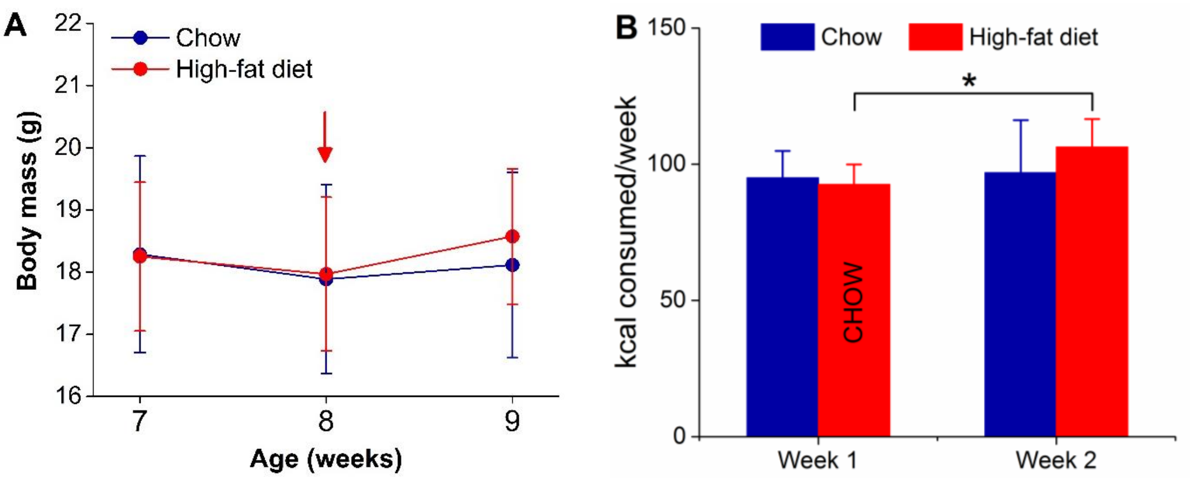

Figure S2. Body mass and food intake in intact female mice (Experiments II and III). Heterozygous PER2::LUC female mice were singly housed in 12L:12D at 7 weeks old and fed chow for 1 week. Beginning at 8 weeks old, mice were fed either chow (n=7; blue symbols) or 45% high-fat diet (n=8; red symbols) for 1 week. Mean (±SD) body mass (A) and food intake (B) were measured weekly. Note that during week 1 (7-8 weeks old), both groups were fed chow. Total kcal consumed was calculated based on the grams of food consumed (chow: 3.02kcal/g metabolizable energy; 45% HFD: 4.73kcal/g). *p<0.05.

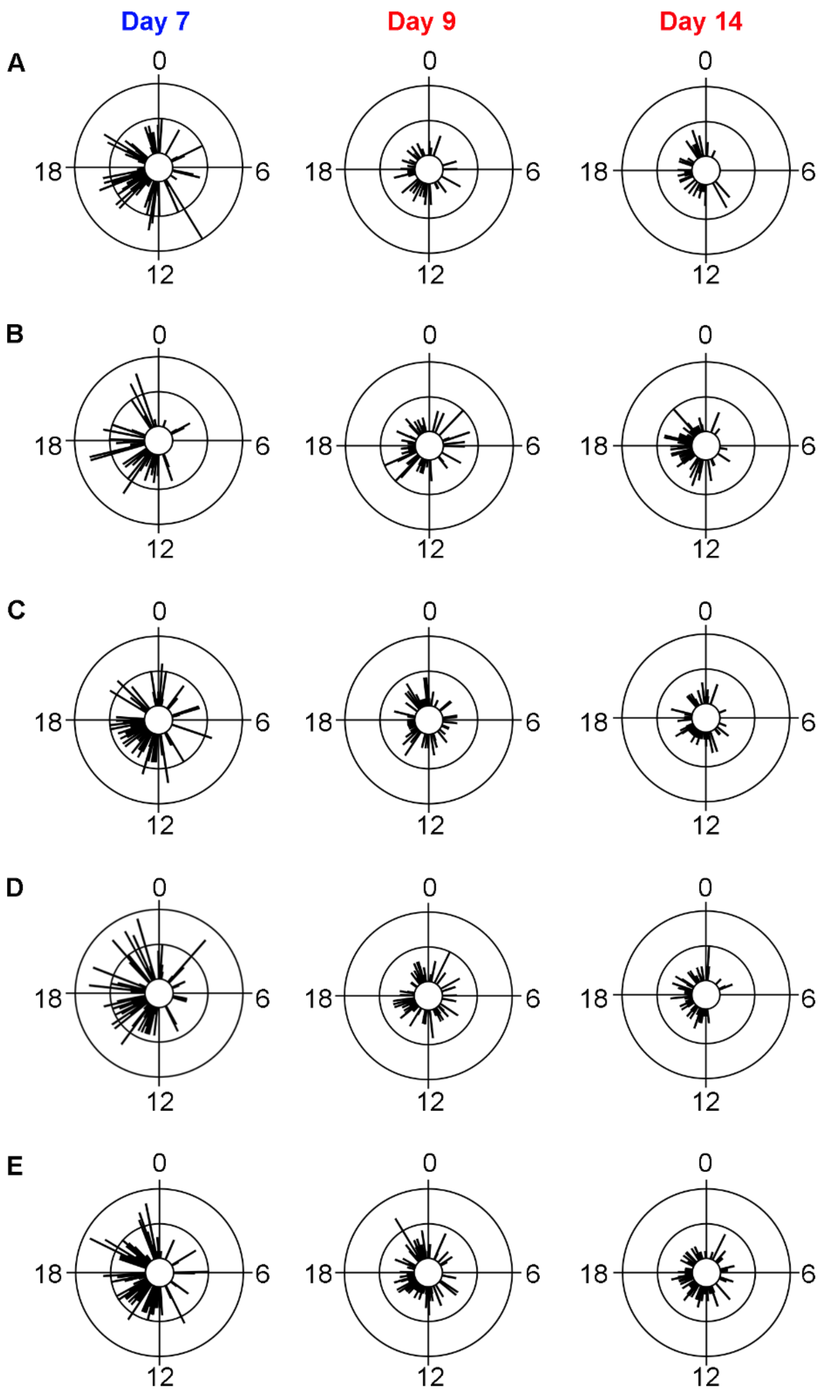

Figure S3. Daily rhythms of eating behavior in intact females during chow and high-fat diet consumption. Circular histograms of eating behavior (10-min bins) in individual mice (A-E). Intact female mice were fed chow (days 1-7) and then high-fat diet (days 9-14). Scale: inner circle, 0; middle circle, 5; outer circle, 10. Lights were on from 0-12.

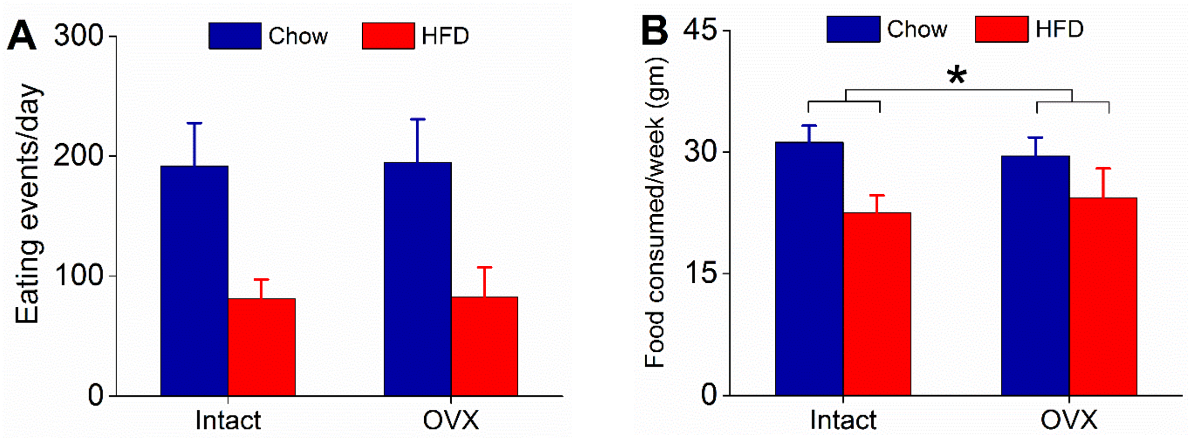

Figure S4. Homeostatic regulation of food intake in intact and ovariectomized female mice. A. The number of eating events (mean±SD) was determined from video recording of chow (day 7) and high-fat (day 14) feeding from intact and ovariectomized (OVX) mice shown in figures 3, S3 and 6, S7, respectively. There was no significant interaction of ovariectomy and diet (F=.003, p=0.96) on the number of eating events. There was a significant main effect of diet (F=74; p<0.001) on the number of eating events (A). **B.** The amount of food consumed per week in grams (mean±SD) during 1 week of chow followed by 1 week of high-fat feeding (same mice as shown in Fig. S2). There was a significant interaction of ovariectomy and diet (F=11, p=0.02) and a main effect of diet (F=56; p<0.001) on the grams of food consumed per week. Compared to chow, intact (p<0.01) and ovariectomized (p=0.01) mice ate significantly fewer grams of high-fat diet. *$p\leq$0.01

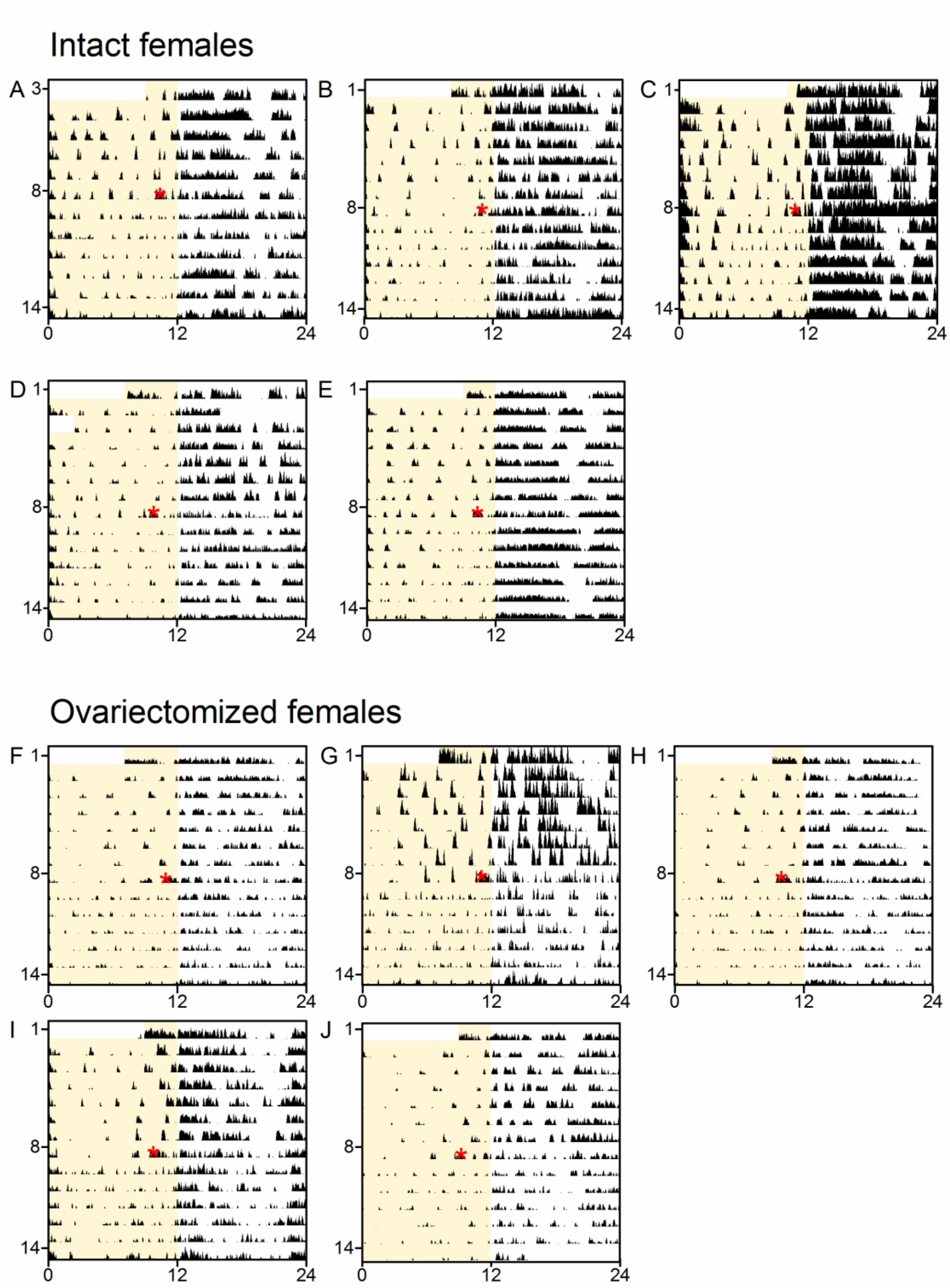

Figure S5. Locomotor activity rhythms during chow and high-fat feeding in female mice. Actograms of locomotor activity in individual intact female (A-E) and ovariectomized female (F-J) mice plotted in 6-min bins (scaled setting in Clocklab, maximum: 8 counts/bin). Chow was replaced with high-fat diet (HFD; red asterisk) on day 8. x-axis: time (hours); y-axis: days. E is shown in Fig. 4A and I is shown in Fig. 7A.

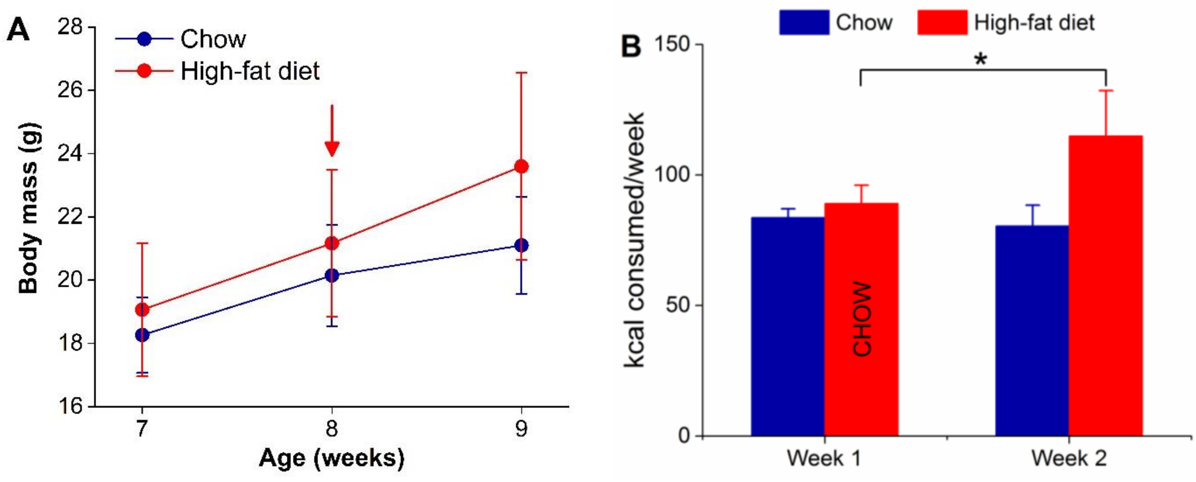

Figure S6. Body mass and food intake in ovariectomized female mice (Experiment III). Heterozygous PER2::LUC female mice were ovariectomized at 6 weeks old, single-housed and fed chow. Beginning at 8 weeks old, mice were fed either chow (n=6; blue symbols) or 45% high-fat diet (n=6; red symbols) for 1 week. Mean (±SD) body mass (A) and food intake (B) were measured weekly. Note that during week 1 (7-8 weeks old), both groups were fed chow. Total kcal consumed was calculated based on the grams of food consumed (chow: 3.02kcal/g metabolizable energy; 45% HFD: 4.73kcal/g). *p<0.01.

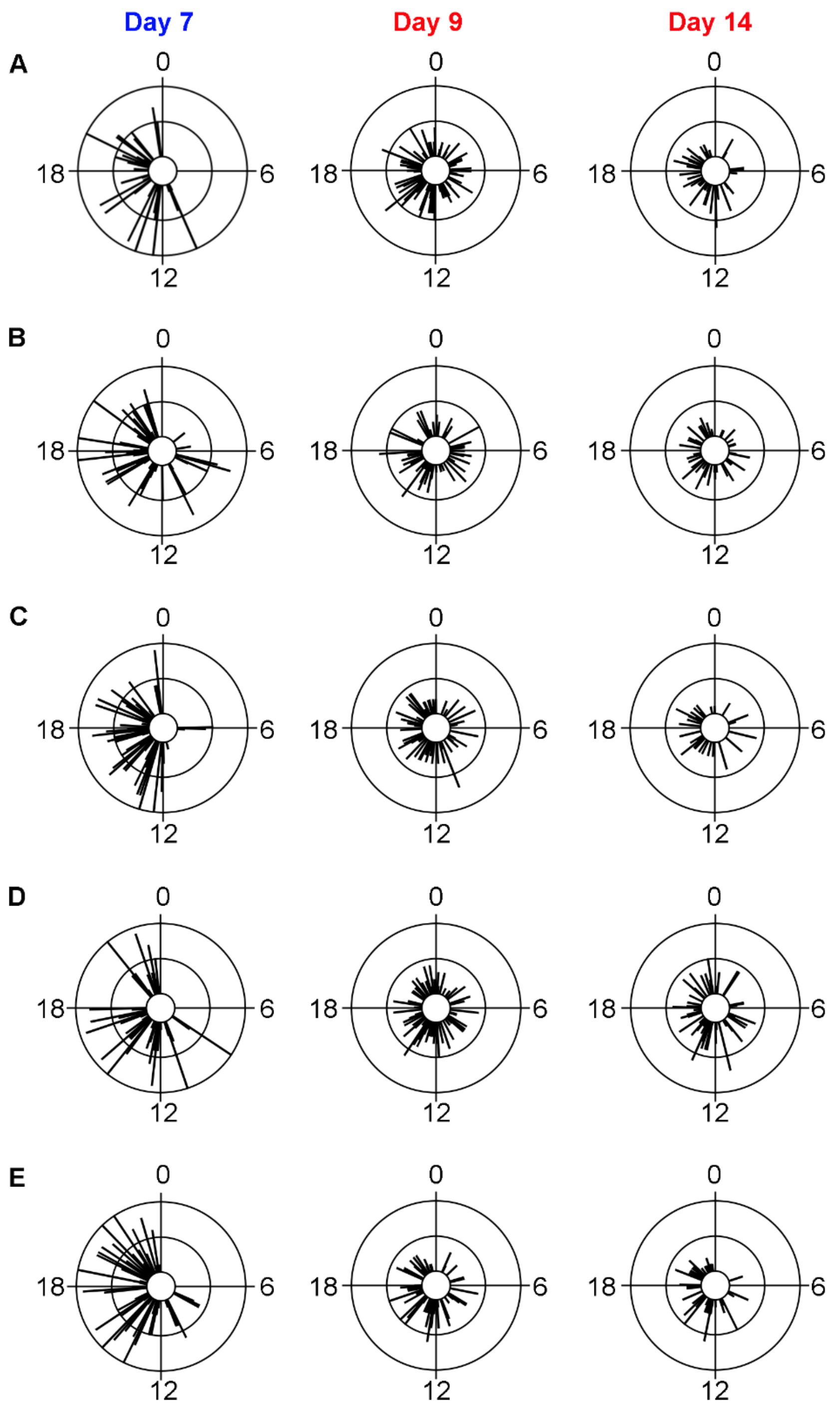

Figure S7. Daily rhythms of eating behavior in ovariectomized females during chow and high-fat diet consumption. Circular histograms of eating behavior (10-min bins) in individual mice (A-E). Female mice were ovariectomized, fed chow (days 1-7) and then high-fat diet (days 9-14). Scale: inner circle, 0; middle circle, 5; outer circle, 10. Lights were on from 0-12.

Table S1. Vector properties of eating behavior rhythms in individual mice.

		**Chow (Day 7)**			**High-fat diet (Day 14)**		
	**Mouse ID**	**Mean angle(μ) ± SD**	**Length (r)**	***P****	**Mean angle (μ) ± SD**	**Length (r)**	***P****
Intact	A	245±79	0.39	Z=30, *p*< 1E-12	272±78	0.40	Z=10, *p*< 7E-05
	B	251±61	0.57	Z=42, *p*< 1E-12	263±58	0.60	Z=35, *p*<1E-12
	C	241±81	0.37	Z=26, *P*=3E-12	245±83	0.35	Z=9, *p*=1E-04
	D	271±73	0.44	Z=40, *p*< 1E-12	269±75	0.42	Z=14, *p*= 1E-06
	E	257±74	0.43	Z=43, *p*< 1E-12	239±87	0.32	Z=10, *p*=6E-05
	Grand mean vector†	253 (226-278)	0.43	F=57 *p*=.004	259 (213-282)	0.41	F=34, *p*= 0.009
OVX	F	247±65	0.53	Z=40, *p*<1E-12	234±77	0.40	Z=13, *p*=3E-06
	G	264±77	0.41	Z=31, *p*<1E-12	231±96	0.23	Z=4, *p*=.01
	H	247±60	0.58	Z=73, *p*< 1E-12	247±85	0.32	Z=6, *p*= .001
	I	237±32	0.46	Z=38, *p*< 1E-12	223±100	0.22	Z=6, *p*=.002
	J	263±66	0.52	Z=64, *p*< 1E-12	239±69	0.44	Z=19, *p*= 7E-09
	Grand mean vector†	251 (229-277)	0.49	F=96, *p*=0.002	263 (198-250)	0.33	F=24, *p*= .01

The mean angle (μ) ± circular standard deviation (SD) and vector length (r) are reported are for individual mice. *Rayleigh's Uniformity test was used to determine if the eating events of individual mice had a significant non-uniform direction (for individual mice). †Hotelling's one sample test was used to test if there was a significant mean direction (for grand mean vectors). The 95% confidence intervals are reported (in parentheses) for the directions of the grand mean vectors. Mouse D is shown in Fig. 3B-D and Mouse I is shown in Fig. 6B-D.
